# From waste to resilience: fish waste priming attenuates drought effects on cucumber seedlings via heightened osmoprotectants, antioxidant activity and metabolic stability

**DOI:** 10.1186/s12870-025-07568-6

**Published:** 2025-11-27

**Authors:** Reda E. Abdelhameed, Rania S. Shehata

**Affiliations:** 1 Botany and Microbiology Department, Faculty of Science, Zagazig University, Zagazig, 44519 Egypt; 2 Biology Department, Faculty of Science, Jazan University, Jizan, 45142 Saudi Arabia

**Keywords:** Priming, Fish waste, Drought, Antioxidant, Biostimulants, Osmolytes, Reactive oxygen species, Sustainable agriculture

## Abstract

Global climate change is intensifying water scarcity, making drought stress a critical challenge for crop production. This study evaluated the potential of fish waste extract (FWE), a circular bioeconomy product, to enhance drought tolerance in cucumber seedlings. A 2 × 4 factorial design (FWE priming × polyethylene glycol at 0, 1, 2, and 3% to simulate drought) was applied to evaluate germination, growth, oxidative stress, antioxidant responses, and secondary metabolites. Qualitative phytochemical screening of FWE revealed the presence of phenolics, amino acids, flavonoids, alkaloids, reducing sugars and coumarins. Drought-stressed cucumber seedlings exhibited reduction in germination parameters and vigor indices, disruption in water homeostasis, decline in membrane stability alongside elevating malondialdehyde (MDA) and hydrogen peroxide (H_2_O_2_) indicating oxidative damage. However, FWE priming significantly alleviated drought effects, enhancing germination, restoring water content and membrane stability, and reducing MDA and H_2_O_2_. Most obviously, under drought stress, FWE amplified proline accumulation and antioxidant enzyme activity, while boosting phenolics and flavonoids reflecting induction of secondary metabolism. Multivariate analyses, including hierarchical clustering and principal component analysis, revealed that FWE treatment significantly enhanced drought tolerance in cucumber seedlings under PEG-induced stress. These findings highlight FWE as a sustainable, low-cost biostimulant that converts agro-industrial byproducts into valuable inputs for drought-resilient cucumber cultivation.

## Introduction

Plants, being sessile organisms, cannot move to avoid harsh surroundings and are constantly exposed to abiotic and biotic stresses that result in large crop losses that threaten global food security [1]. Among these, drought stress, the lack of water availability in both amount and distribution over the course of a plant’s life cycle, is a major abiotic stressor that impacts plant and agronomic productivity globally [2, 3]. Since water is an essential component of cells and a basic material for plant cell division and growth, plant growth and development are extremely sensitive to water shortages [4, 5]. Reports indicate that drought leads to severe dehydration of the protoplasm, thereby disrupting photosynthesis, respiration, translocation, ion absorption, nutrient metabolism, and various other physiological and biochemical processes in plants [2, 4, 6]. To counteract these effects, plants activate defense mechanisms involving osmoprotectants, antioxidants, and ROS-scavenging enzymes [1, 7].

Developing effective and low-cost approaches to enhance drought tolerance is therefore a priority. Conventional reliance on chemical fertilizers can partially mitigate stress but raises concerns about soil, water, and ecosystem health. Sustainable alternatives are urgently needed to reduce agrochemical dependence while maintaining productivity under water scarcity [8].

Biostimulants derived from natural sources have emerged as promising solutions [9, 10]. Compounds such as seaweed extracts, microbial inoculants, and fish waste derivatives are rich in nutrients and bioactive molecules that promote phytohormone regulation, osmotic adjustment, and antioxidant activity [2, 10–12]. It is often believed that biostimulants’ capacity to deliver an exogenous supply of macro- and micronutrients is what enables them to significantly boost plant growth in response to stresses [13].

Fish waste in particular is an abundant underutilized byproduct of fisheries and processing industries. It contains proteins, amino acids, lipids, and minerals such as nitrogen, phosphorus, and calcium, all of which can support plant growth and stress adaptation. It is estimated that around 75% of the total fish weight, including bones, heads, viscera, skin, and fins, is discarded as waste [14, 15]. These fisheries and markets often lack basic waste management systems, resulting in much of this material, both biodegradable and non-biodegradable, not being collected and instead dumped as garbage, despite its potential nutritional value. Repurposing fish waste into agricultural inputs also aligns with circular economy principles by transforming environmental dangers into valuable bioproducts [16, 17].

Seed priming with natural extracts offers a simple and effective strategy to improve germination and seedling vigor under stress [18, 19]. Priming initiates early metabolic processes, enhances antioxidant defenses, and strengthens the seed’s capacity to withstand unfavorable conditions [20]. Prior studies have shown that priming can increase activities of peroxidase, superoxide dismutase, and other protective systems, resulting in improved emergence and stress resilience [21–23].

Based on these insights, the present study investigates the potential of fish waste extract (FWE) priming to enhance cucumber seedling performance under drought simulated by polyethylene glycol (PEG). Specifically, we assess germination, seedling growth, water status, oxidative stress markers, antioxidant enzyme activity, and accumulation of proline and secondary metabolites. The study highlights the valorization of fish waste as a sustainable biostimulant within a circular bioeconomy framework, offering a practical strategy to improve crop drought tolerance.

## Materials and methods

### Setting up fish waste powder

The fish and shrimp wastes were collected, rinsed with detergent after being repeatedly cleaned with flowing warm water to remove any soluble organic materials and other contaminants. Following seven days of drying at 50 °C in an oven, the cleaned waste was crushed and sieved to a fine powder. Then, the powdered fish waste was put in plastic bags and kept at room temperature until it was needed.

### Fish waste’s nutritional composition

The nutritional content of fish waste was analyzed following digestion and mineralization using concentrated sulfuric acid, with hydrogen peroxide serving as a catalyst. The analysis was conducted at the Central Laboratory, Faculty of Agriculture, Zagazig University, Egypt. Additionally, the amino acid profile of the powdered fish waste was determined using a high-performance amino acid analyzer (Biochrom 30) at the Regional Food and Feed Center, Agricultural Research Centre, Giza, Egypt as previously mentioned in our previously published paper of Metwally et al**.** [12].

### Preparation of FWE and its phytochemical analysis

The powdered fish waste was weighed and suspended in water at a 10% (w/v) ratio (10 g powder per 100 mL water), homogenized with constant stirring for 24 h at room temperature, and filtered through Whatman No. 1 paper. The filtrate was adjusted to neutral pH (≈ 7.0). The extract was freshly prepared under aseptic conditions to ensure sterility and avoid microbial contamination.

The phytochemical screening of the prepared 10% (w/v) FWE was done observing the procedures outlined by Yadav et al. [24], Iqbal et al. [25] and Shaikh & Patil [26] for identifying various phytochemical components. To test for phenols, 1 mL of FWE was mixed with 2 mL of distilled water, followed by the addition of a few drops of 10% ferric chloride solution. The development of a blue or green color showed the existence of phenolic compounds. Flavonoids were identified by mixing 1 mL of FWE with 1 mL of 2 N NaOH solution, where the formation of a yellow precipitate confirmed their presence. For tannins, equal volumes of distilled water and FWE were combined, stirred, and then treated with a few drops of ferric chloride; a green precipitate signified the presence of tannins. Alkaloids were detected by adding Wagner’s reagent to a mixture of 3 mL of FWE and 3 mL of 1% HCl, heated in a steam bath. The resulting turbidity confirmed their presence. To detect coumarins, 1 mL of FWE was added to 1 mL of 10% NaOH solution, with the appearance of a yellow color indicating their presence. For saponins, 5 mL of FWE and 5 mL of distilled water were vigorously shaken and heated in a test tube; the formation of stable foam confirmed their presence.

### Seeds, treatments and experimental conditions

Cucumber seeds (*Cucumis sativus* L.) (HAYEL) were obtained after permission from the Ministry of Agriculture, Zagazig, Egypt, then were treated as experimental materials. Uniform seeds were surface-sterilized using 5% sodium hypochlorite for 10 min, followed by thorough rinsing with sterilized distilled water. After sterilization, the seeds were divided into two groups; the first group was unprimed (control) and the second group was soaked in 10% fish waste extract (FWE). Seeds were soaked for 12 h at 25 °C in the dark then left to dry in air for 6 h.

Each group’s seeds were germinated in two filter paper folds that were put in Petri dishes with a diameter of 12 cm and 10 milliliters of the test solutions. For each treatment, five petri dishes (*n* = 5) were used and each petri dish has 20 seeds. After introducing inactive osmotica and non-penetrating polymers of polyethylene glycol (PEG 6000), seeds were allowed to germinate under four different levels of water stress; at the level of 0, 1, 2 and 3% (w/w) equivalent to osmotic potential 0, −0.00756, −0.0174 and − 0.0297 MPa (Megapascal) respectively (Table 1) which calculated based on the equation below [27].

**Table 1:** Calculated osmotic potentials (Ψ) of PEG-6000 solutions (1, 2 and 3% w/w) at 20 °C using the Michel and Kaufmann [27] equation

% (w/w) PEG	C (g·kg⁻¹ water)	Ψ (bar)	Ψ (MPa)
0%	-	-	-
1%	10.1010	−0.07558	−0.007558
2%	20.4082	−0.17399	−0.017399
3%	30.9278	−0.29661	−0.029661

$$\begin{aligned} \:{{\uppsi\:}}_{sol}\left(\text{b}\text{a}\text{r}\right)=&-1.8\:\text{x}\:{10}^{-2}\:\text{C}\:-\:1.18\:\text{x}\:{10}^{-4}\:{\text{C}}^{2}\:\\&+\:2.67\:\text{x}\:{10}^{-4}\text{C}\text{T}\:+\:8.39\:\text{x}{10}^{-7}{\text{C}}^{2}\text{T} \end{aligned}$$

Ψ_sol_​ is osmotic potential in bar (1 bar = 0.1 MPa), C = PEG-6000 concentration in g PEG per kg H_2_​O (g·kg^−1^), and T = temperature 20 °C.

So, eight treatments were obtained (2 priming X 4 PEG levels). Experiments on germination were carried out at 20 °C in growth conditions. Seedlings from each treatment were taken out after 12 days, where the visual differences between treatments were more obvious, to measure the germination parameters, and the remaining seedlings were powdered in liquid nitrogen and kept at −80 °C for additional examination.

###  Monitoring of germination dynamics

Radicle visibility was used to determine when a seed had germinated. Following ISTA guidelines, seedlings were picked, and observations were made about their growth [28]. Shoot length (SL, in cm) and radicle length (RL, in cm) was assessed using randomly selected seedlings using meter scale. The seedling fresh weight (SFW, g) and seedling dry weight (SDW, in g) were documented after oven drying at 65 °C for 72 h. Moreover, the percent of improvement of FWE priming for each germination parameter (GP) under different drought was calculated as follows:

$$\begin{aligned} \:\text{F}\text{W}\text{E}\:\text{I}\text{m}\text{p}\text{r}\text{o}\text{v}\text{e}\text{m}\text{e}\text{n}\text{t}\:\left(\%\right)=\frac{\:\text{G}\text{e}\text{r}\text{m}\text{i}\text{n}\text{a}\text{t}\text{i}\text{o}\text{n}\:\text{p}\text{a}\text{r}\text{a}\text{m}\text{e}\text{t}\text{e}\text{r}\:\left(\text{G}\text{P}\right)\:\text{o}\text{f}\:\text{F}\text{W}\text{E}\:\text{t}\text{r}\text{e}\text{a}\text{t}\text{e}\text{d}\:\text{p}\text{l}\text{a}\text{n}\text{t}\text{s}-\:\text{G}\text{P}\:\text{o}\text{f}\:\text{c}\text{o}\text{n}\text{t}\text{r}\text{o}\text{l}\:\text{p}\text{l}\text{a}\text{n}\text{t}}{\:\:\:\:\:\text{G}\text{P}\:\text{o}\text{f}\:\text{c}\text{o}\text{n}\text{t}\text{r}\text{o}\text{l}\:\text{p}\text{l}\text{a}\text{n}\text{t}\text{s}}\times100 \end{aligned}$$

Regarding seedling vigor indices: These indices were proposed by Abdul-Baki and Anderson [29], and are calculated based on germination and seedling growth parameters, such as seedling length and DW. The formulas are as follows:

$$\begin{aligned} \mathrm{Germination}\;\mathrm{percentage}\;(\%)\;=&\;(\mathrm{number}\;\mathrm{of}\;\mathrm{normally}\;\mathrm{germinated}\;\mathrm{seeds}\\&/\mathrm{total}\;\mathrm{number}\;\mathrm{of}\;\mathrm{seeds}\;\mathrm{for}\;\mathrm{testing})\;\times\;100\% \end{aligned}$$

$$\mathrm{Vigor}\;\mathrm{index}-\mathrm I\:=\:\mathrm{Germination}\;(\%)\;\times\;\mathrm{Mean}\;\mathrm{seedling}\;\mathrm{length}\;(\mathrm{cm})$$

$$\mathrm{Vigor}\;\mathrm{index}-\mathrm{II}\:=\:\mathrm{Germination}\;(\%)\;\times\;\mathrm{Mean}\;\mathrm{seedling}\;\mathrm{DW}\;(\mathrm{mg})$$

### Determination of water status and membrane stability index (MSI)

Water status comprising relative water content (RWC) and water saturation deficit (WSD) were estimated in cucumber seedlings [30] and calculated using the following formulas:

$$\mathrm{RWC}\;(\%)\;=\frac{(\mathrm{FW}\;-\;\mathrm{DW})}{(\mathrm{TW}\;-\;\mathrm{DW})}\mathrm X\;100$$

$$\:\text{W}\text{S}\text{D}\:\left(\%\right)=\:100-\text{R}\text{W}\text{C}$$

* FW, DW and TW represent the fresh, dry and turgid weights; respectively.

For MSI determination, ten mL of deionized water and 0.1 g of fresh cucumber seedling were mixed in test tubes. These samples were prepared in two similar sets. After one set was submerged in a water bath at 40 °C for 30 min, electrical conductivity (EC) was measured using an EC meter and found to be C1. The electrical conductivity was found to be C2 after the second set of tubes was heated in a water bath for 30 min at 100 °C. MSI was considered using the formula provided by Farooq and Azam [31] as follow:

$$\mathrm{MSI}\;(\%)\;=\;\lbrack1\;-\;(\mathrm C1/\mathrm C2)\rbrack\;\times\;100$$

Membrane injury (MI) was intended using Dhanda et al. [32] as the ratio of drought-stressed plants’ MSI (MSI_d) to control plants’ MSI (MSI_c).

$$\mathrm{MI}(\%)=\lbrack1-({\mathrm{MSI}}_{\mathrm d}/{\mathrm{MSI}}_{\mathrm c})\rbrack\times100$$

## Oxidative stress marker (H_2_O_2_ and lipid peroxidation)

The H_2_O_2_ content was calculated using the methodology outlined by Velikova et al. [33]. 0.1% trichloroacetic acid (TCA) was used to homogenize the weight of fresh cucumbers. After that, 0.5 ml of the extract was mixed with 0.5 ml of 100 mM K-phosphate buffer (pH 6.8) and 2 ml of 1 M KI, and the mixture was left to incubate for one hour in the dark. At 390 nm, the absorbance was measured in relation to a reagent blank. A standard calibration curve created by varying H_2_O_2_ concentrations was used to measure the H_2_O_2_ (µmol g^−1^ FW) content.

The thiobarbituric acid method was used to measure the production of malondialdehyde (MDA) in order to detect lipid peroxidation [34]. The absorbance was measured at 530 nm and subtracted from the absorbance at 600 nm to account for non-specific turbidity. To calculate the MDA concentration (nmol g^−1^ FW), the extinction coefficient of 155 mM^−1^ cm^−1^ was used.

### Osmolytes determination

Free proline content was estimated following the method described by Bates et al. [35]. Fresh cucumber tissue was homogenized in 10 mL of 3% (w/v) aqueous sulfosalicylic acid. Two milliliters of the resulting filtrate were mixed with 2 mL of glacial acetic acid and 2 mL of acid ninhydrin in a glass test tube, then incubated in a boiling water bath for 1 h. The reaction was terminated by placing the tubes in an ice bath. Subsequently, 4 mL of toluene were added to each tube, and the mixture was stirred vigorously for 20–30 s. The upper toluene layer, containing the chromophore, was separated, and its absorbance was measured at 520 nm using a UV-Visible spectrophotometer (RIGOL, Model Ultra-3660).

Total soluble protein content was determined using the method of Lowry et al. [36]. Fresh cucumber seedling tissue was homogenized in 5 mL of phosphate buffer. One milliliter of the extracted protein solution was mixed with 5 mL of freshly prepared alkaline reagent. After 10 min, 0.5 mL of diluted Folin–Ciocalteu reagent was added. The mixture was left to stand for 20 min, and the absorbance was then measured at 700 nm. Protein concentration was calculated based on a standard curve prepared using bovine serum albumin.

### Secondary metabolites estimation

Calculating the overall flavonoid content (TFC) in plants is typically done using the colorimetric approach for aluminum chloride (AlCl_3_) [37]. This procedure involves mixing plant extracts with AlCl_3_ and the formation of chelates of Al(III)-flavonoids a yellow complex. The flavonoid concentration is then determined using a standard curve, frequently based on quercetin, after the color’s intensity is measured at 415 nm.

Using gallic acid as a reference, the Folin–Ciocalteu test was used to spectrophotometrically measure the total phenolic content (TPC) in the methanolic leaf extract using a UV-visible spectrophotometer [RIGOL, Model Ultra-3660] [38]. We can determine the extracts’ total phenolic contents by applying the following formula:

$$\mathrm C=\mathrm{cV}/\mathrm W$$

where W is the weight of cucumber tissue, V is the volume of extract in milliliters, c is the concentration of gallic acid determined from the calibration curve, and C is the TPC in µg g^−1^ in gallic acid equivalent.

### Assays of antioxidant enzyme activity

Fresh cucumber samples were gathered in order to measure the activity of antioxidant enzymes. The homogenized seedlings were extracted using potassium phosphate buffer (pH 6.8, 10 mM) containing 1% polyvinyl pyrrolidone using a magnetic stirrer for 10 min. All enzyme extraction procedures were performed at 4 °C. The homogenate was centrifuged for 20 min at 6000 rpm using centrifuge (Gemmy PLC-03) and the supernatant was gathered for catalase (CAT), peroxidase (POX), ascorbate peroxidase (APX) and polyphenol oxidase (PPO) assays which expressed as U g^−1^ FW min^−1^. CAT activity was carried out in fresh supernatant (0.5 mL) in accordance with Aebi’s [39] instructions. The procedure proposed by Maehly and Chance [40] was used to measure POX activity. At 290 nm, the activity of APX was measured using the Nakano and Asada [41] technique while at 495 nm absorbance, the Lavid et al. [42] approach was done to determine the activity of the PPO.

### Phosphomolybdate assay for the total antioxidant capacity (TAC)

 Prieto et al. provided a description of TAC [43]. Cucumber methanolic extract was combined with 3 mL of a reagent consisting of 0.6 M sulfuric acid, 28 mM sodium phosphate Na_2_HPO_4_, and 4 mM ammonium molybdate. The mixture was then incubated for 90 min at 90ᴼC in a water bath, where antioxidant compounds reduce Mo(VI) to Mo(V), forming a green phosphate/Mo(V) complex. After the samples had cooled to room temperature, the absorbance of the solution was measured at 695 nm against a blank. The blank solution was prepared by mixing 3 mL of the reagent solution with the corresponding volume of the methanol and incubated under the same conditions as the test samples. The results are typically expressed as ascorbic acid equivalents (mg g^−1^ FW) by preparing a standard calibration curve of a series of ascorbic acid at known concentrations (0.01–1.0 mg mL^−1^).

### Statistical data processing

The effects of FWE priming and varying PEG concentrations on cucumber plants were analyzed using one-way analysis of variance (ANOVA) in the SPSS software (Statistical Package for Social Science version 16.0). For each treatment, 20 seeds were placed per plate with five replicate plates (*n* = 5). From each plate, ten randomly selected normal seedlings were measured, and the recorded values were averaged to obtain a single mean per plate. These plate means were used for statistical analyses, with the five plates serving as biological replicates. Results were expressed with their corresponding standard errors. Treatment means were compared using Duncan’s Multiple Range Test at a significance level of 0.05. Different lowercase letters were used to indicate statistically significant differences among treatments. Two-way ANOVA was conducted to evaluate the effects of FWE priming and PEG stress as fixed factors and their interactions for germination, physiological and biochemical parameters followed by Duncan’s test for post-hoc comparisons. Additionally, correlation analysis, hierarchical clustering (HC), and principal component analysis were performed using the software PAST (PAleontological STatistics version 4.03) to explore relationships among parameters and treatment groupings. HC was performed using Euclidean distance and paired group (UPGMA) method, enabling the identification of treatment groups with similar responses.

## Results and discussion

Many seed treatments, including hydration pre-sowing, seed coating with bio-control agents, and bio-priming seed treatments, have emerged as significant and environmentally friendly substitutes for the current fungicide seed treatment in recent years [44]. It is a challenge to reduce drought stress in this way by employing a seed treatment strategy based on novel bioactive chemicals [45].

In this study FWE was prepared and its phytochemical analysis (Table 2) indicated the existence of several phyto-constituents such as phenolics, amino acids, flavonoids, alkaloids, reducing sugars and coumarins. Also, FWE contained macro and micronutrients as demonstrated by our earlier recorded data [12] where the findings demonstrated that FWE is abundant in Zn (94.37 µg/g), Mg (189 µg/g), Fe (266.81 µg/g), Ca (89,762.5 µg/g), K (1573 µg/g), P (12,201 µg/g), and N (7.01%). Moreover, FWE is rich with various essential and non-essential amino acids as earlier mentioned in our studies [12, 46]. There are seventeen amino acids present in the fish waste analysis as revealed in our previous study by Metwally et al. [12]. From which, aspartic acid (2.79%), glycine (3.13%), and glutamic (4.28%) are the uppermost three amino acids, while methionine (0.57%), histidine (0.55%), and cysteine (0.2%) are the lowermost three. Meanwhile, the remaining eleven amino acids (alanine, proline, arginine, valine, leucine, phenylalanine, serine, lysine, tyrosine, threonine and isoleucine) were in-between in their concentration (Table 3).

**Table 2:** Qualitative phytochemicals screening of fish waste extract (FWE)

Phytochemicals	FWE
Phenols	++
Flavonoids	++
Reducing sugars	++
Alkaloids	+
Protein	+
Coumarins	++
Tannins	
Saponins	+
Amino acids	+

**Table 3:** Amino acids analysis of fish wastes

Amino acid	%	Amino acid	%
Aspartic (ASP)	2.19	Tyrosine (TYR)	1.06
Therionine (THR)	1.08	Phenylalanine (PHE)	1.21
Serine (SER)	1.19	Histidine (HIS)	0.55
Glutamic (GLU)	3.28	Lysine (LYS)	1.18
Glycine (GLY)	4.13	Argnine (ARG)	1.77
Alanine (ALA)	2.58	Proline (PRO)	2.53
Valine (VAL)	1.30	Cystine (CYS)	0.20
Isoleucine (ILE)	0.71	Methionine	0.57
Leucine (LEU)	1.28		

These findings align with those of Susitha and Thiripurasundari [47], who reported that fish waste is rich in nutrients beneficial for plant growth. In this context, the present study explores the potential of fish waste as a valuable resource by utilizing its extract (FWE) as a biostimulant and seed priming agent to enhance germination parameters, framed within the concept of a circular bio-economy.

### Priming with FWE improved cucumber germination and seedling parameters under PEG-induced water deficit conditions

**Fig. 1:**
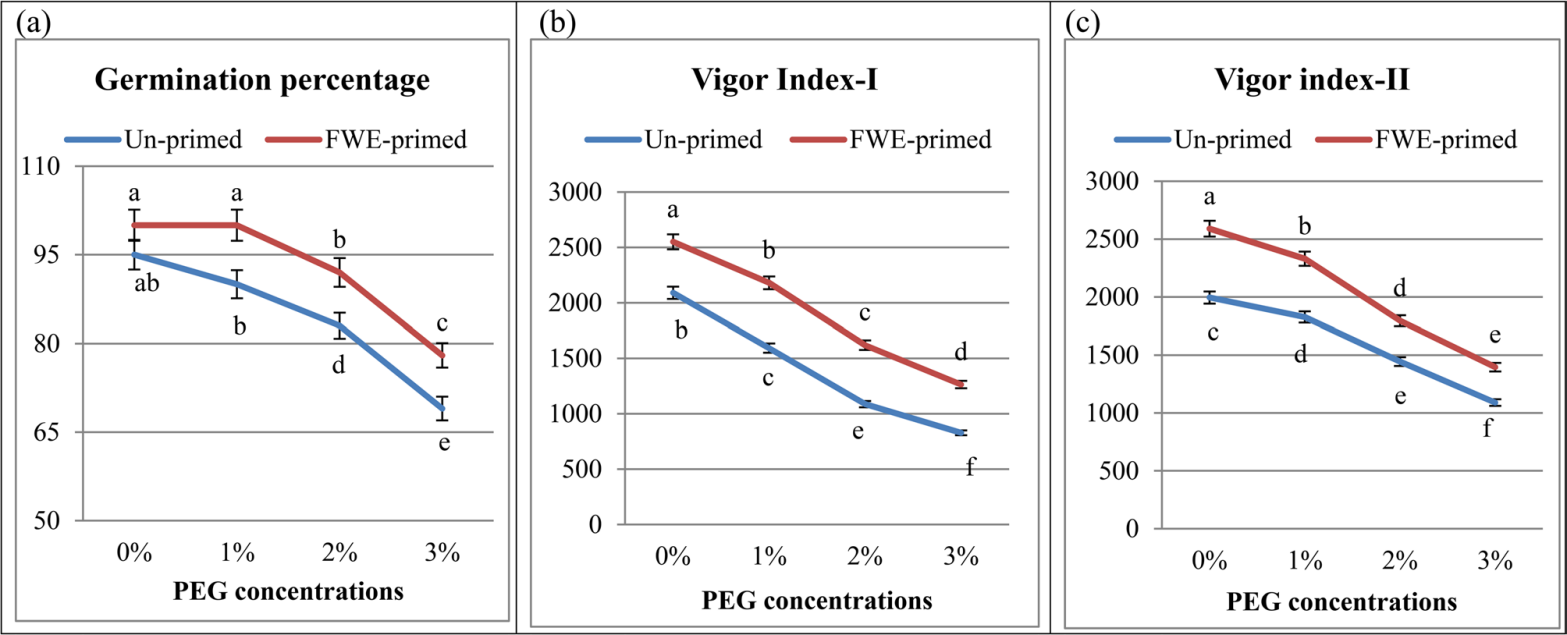
Influence of priming with fish waste extract (FWE) on germination percentage (**a**), vigor index-I (**b**) and vigor index-II (**c**) of cucumber seedlings under drought stress (different polyethylene glycol: PEG). Across all treatments, data are means (n = 5) ± standard errors (error bars), columns followed by the same letters do not differ statistically by Duncan’s multiple range tests (Level 0.05)

The response of cucumber for seed treatments (FWE) under different PEG concentration were interpreted in terms of seedling FW, seedling DW, seedling length, radicle length and shoot length. Under PEG treatments (drought stress), the phenotypic appearance (germination and seedling parameters) of cucumber plants was negatively affected compared to the control conditions (Table 4 and Fig. 1). Drought stress (3% PEG) significantly impaired cucumber seed germination, manifesting as a reduced seedling total length (12 ± 0.635e), FW (0.18 ± 0.0095c) and DW (0.0158 ± 0.00088c) relative to 22 ± 1.111ab, 0.28 ± 0.0148b and 0.0216 ± 0.0011b of control, respectively. These findings align with prior studies where water scarcity disrupts imbibition and metabolic reactivation, critical for radicle emergence [4]. Consistent with our findings, Mickky and Aldesuquy [48] reported that PEG-induced drought stress adversely affected the morphological traits of wheat seedlings, including reductions in plumule and radicle length, seedling FW, water content, and the number of adventitious roots. Similarly, Ma et al. [49] demonstrated that PEG-6000-induced water deficiency significantly reduced seed germination rates, with no germination observed at 20% PEG concentration. Besides, water stress conditions led to notable morphological alterations in both seedling leaves and roots due to a pronounced decline in the environmental water potential [44].

**Table 4:** Mean performance of cucumber seedling growth parameters and percent improvement (%) as influenced by seed priming with fish waste extract (FWE) under drought stress (different polyethylene glycol: PEG)

Treatments		Germination parameters									
PEG conc. %	Priming	Shoot length (SL), cm	%	Radicle length (RL), cm	%	Seedling total length, cm	%	Seedling fresh weight (SFW), g	%	Seedling dry weight (SDW), g	%
0	**Un-primed**	12.0 ± 0.635b	-	10.0 ± 0.476a	-	22.0 ± 1.11ab		0.28 ± 0.0148b		0.0216 ± 0.0011b	
	**FWE-primed**	14.7 ± 0.741a	22.5	10.8 ± 0.479a	8.0	25.5 ± 1.217a	15.9	0.36 ± 0.019a	28.6	0.0259 ± 0.0014a	19.9
1	**Un-primed**	10.0 ± 0.529c	-	7.7 ± 0.370b	-	17.7 ± 0.899 cd		0.25 ± 0.015b		0.0203 ± 0.0011bc	
	**FWE-primed**	12.5 ± 0.634b	25.0	9.3 ± 0.371b	20.7	21.8 ± 1.01bc	23.2	0.32 ± 0.0159b	28.0	0.0233 ± 0.0011bc	14.8
2	**Un-primed**	8.1 ± 0.529c	-	5.0 ± 0.264c	-	13. 1 ± 0.793d		0.22 ± 0.0116c		0.0174 ± 0.0009c	
	**FWE-primed**	10.4 ± 0.528c	29.6	7.2 ± 0.372b	44.0	17.6 ± 0.899 cd	34.3	0.29 ± 0.0148b	31.8	0.0195 ± 0.0011bc	12.2
3	**Un-primed**	7.0 ± 0.370d	-	5.0 ± 0.265c	-	12.0 ± 0.635e		0.18 ± 0.0095c		0.0158 ± 0.00088c	
	**FWE-primed**	9.5 ± 0.423d	35.7	6.7 ± 0.317b	34.0	16.2 ± 0.741de	35.0	0.23 ± 0.0116c	28.7	0.0179 ± 0.00089c	13.3

Means (n = 5) for each parameter (± standard error) marked with different letters are significantly different according to Duncan’s multiple range tests (Level 0.05)

Germination begins with water uptake, which softens and ruptures the seed coat, allowing the radicle and plumule to emerge as the embryo mobilizes stored food reserves through hydrolysis [48]. Insufficient water disrupts key physiological and metabolic processes, including enzyme activity and cell division, leading to reduced seed performance. Limited water availability also lowers seed vigor by restricting imbibition, thereby hindering germination [50, 51]. PEG-6000-induced osmotic stress likely interferes with water uptake during germination, leading to reduced enzyme activity and slower germination rates [52, 53]. Consequently, water scarcity is expected to suppress seed germination and retard seedling growth. This is supported by our findings, where increasing concentrations of PEG resulted in significant reductions in shoot and radicle length, as well as in the fresh and dry weights of cucumber seedlings.

In contrary, the characteristics of seedlings and germination were greatly enhanced by FWE priming as compared to control. Data in Table 4 exhibited a significant variation in seedling parameters of cucumber primed with FWE under different PEG. Among the treatments, FWE treated seeds recorded maximum seedling length (cm: 25.5 ± 1.217a), fresh (g: 0.36 ± 0.019a) and dry weights (g: 0.0259 ± 0.0014a). The characteristics of early and quick germination include longer roots and shoots as well as higher fresh and dry weights of the seedlings raised the seedling vigor indices in FWE treated seeds as highlighted in Fig. (1). In line with this study, a research done by Susitha and Thiripurasundari [47] highlighted the increase in the number of seeds germinated, height of the plant, and level of the minerals in the fenugreek plant upon application of fish waste fertilizer. Also the previous study confirmed the function of fish waste as a superior organic fertilizer that continuously supplies nutrients to the soil, improving the fenugreek’s development and nutritional value and establishing an environmentally and economically viable method of turning waste into wealth.

In PEG (3%) treated seeds, priming with FWE recorded a greater increase (35, 28.7 and 13.3%) in seedling length, fresh and dry weights respectively over the corresponding PEG treated only. The observed variation in seed germination parameters and seedling length may be attributed to the growth-promoting effects of the seed primer (FWE). Upon seed imbibition, FWE may release growth-regulating compounds and essential nutrients, as it is a rich source of amino acids, proteins, carbohydrates, and minerals such as P, Zn, N, Ca, K, Mg, and Fe. These components have been shown to enhance root development and leaf area in onion plants [12, 54]. In support of this, Metwally et al. [12] reported significant improvements in onion growth traits (fresh and dry weight, plant height, root length, leaf number, bulb and neck diameter) following the application of fish waste-based treatments. Our results were also consistent with Ellyzatul et al. [15] and Ranasinghe et al. [55] who found that applying fish waste to *Albemonchus esculentus* and cucumber plants greatly enhanced their leaf number, leaf area, shoot length, and fruit weight. Because fish waste contains chitin, nature’s second most prevalent polysaccharide after cellulose, plants grown on it may grow more easily [56].

### Priming with FWE maintained water homeostasis and membrane stability in cucumber seedlings

**Fig. 2:**
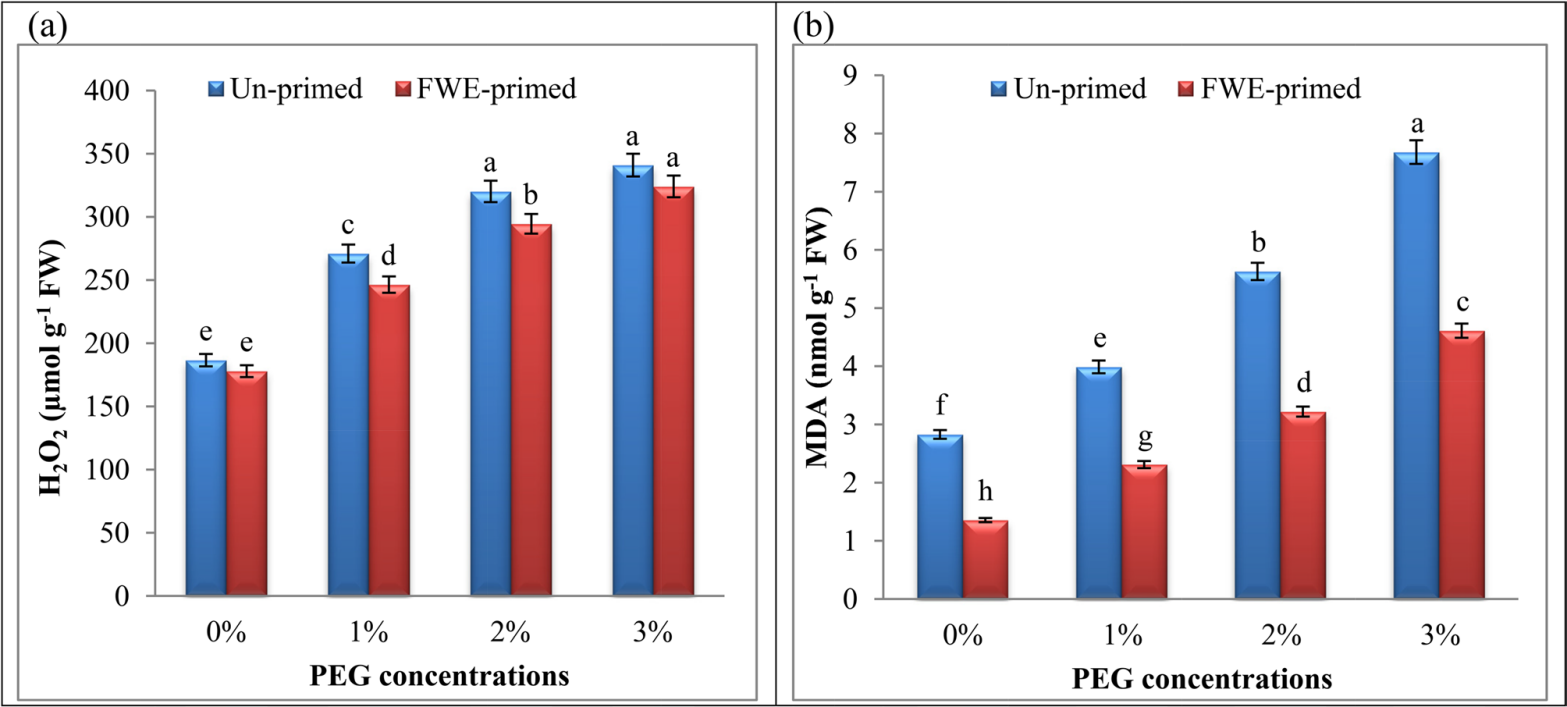
Influence of priming with fish waste extract (FWE) on oxidative stress [(**a**) H_2_O_2_: hydrogen peroxide and (**b**) MDA: malondialdehyde] of cucumber seedlings under drought stress (different polyethylene glycol: PEG). MDA content was estimated following the thiobarbituric acid (TBA) assay, and calculations were based on an extinction coefficient of 155 mM^−1^ cm^−1^. Across all treatments, data are means (n = 5) ± standard errors (error bars), columns followed by the same letters do not differ statistically by Duncan’s multiple range tests (Level 0.05)

Since water status is crucial for plants under drought stress, we assessed relative water content (RWC) and water saturation deficit (WSD) (Table 5). It was found a significant decrease in RWC in PEG-stressed cucumber (88.22, 79.85 and 69.07% at 1, 2 and 3% PEG, respectively). Our findings indicated that treatment with FWE had the highest RWC under non-PEG circumstances. Furthermore, WSD significantly increased under PEG treated cucumber; however, its effect was lessened with FWE application (Table 5). Drought-stressed seedlings showed a reduction in RWC compared to controls, indicative of cellular dehydration [3]. Also, cucumber seedlings experienced a lower MSI values with increasing PEG concentrations. The decrease MSI values may be due to the increased production of ROS and lipid peroxidation (shown later; Fig. 2) of cell membranes leads to disruption of membrane structure, and increased ion leakage indicating compromised membrane integrity.

**Table 5:** Water status (relative water content: RWC and water saturation deficit: WSD), membrane stability index (MSI), membrane injury (MI) and total antioxidant capacity (TAC) of cucumber seedlings as influenced by seed priming with fish waste extract (FWE) under drought stress (different polyethylene glycol: PEG)

PEG conc. %	Priming	RWC %	WSD %	MSI %	MI %	TAC (mg g^−1^ FW)
**0**	**Un-primed**	91.73 ± 4.854a	8.27 ± 0.437d	60 ± 1.59b	-	2.762 ± 0.073f
	**FWE-primed**	93.35 ± 4.939a	6.65 ± 0.352d	67 ± 1.72a	-	3.658 ± 0.096e
**1**	**Un-primed**	88.22 ± 4.668a	11.78 ± 0.623c	51 ± 1.35d	15 ± 0.39e	3.678 ± 0.10e
	**FWE-primed**	90.40 ± 4.89a	9.60 ± 0.402d	56 ± 1.45c	7 ± 0.18f	3.781 ± 0.117e
**2**	**Un-primed**	79.85 ± 4.225ab	20.15 ± 1.066b	42 ± 1.11e	30 ± 0.79c	4.421 ± 0.157d
	**FWE-primed**	87.66 ± 4.85a	12.34 ± 0.441d	49 ± 1.29d	18.3 ± 0.48d	5.51 ± 0.159c
**3**	**Un-primed**	69.07 ± 3.65b	30.93 ± 1.636a	30 ± 0.79 g	50 ± 1.32a	5.91 ± 0.156b
	**FWE-primed**	77.75 ± 4.696a	22.25 ± 0.595c	37 ± 0.97f	38.4 ± 1.02b	7.73 ± 0.204a

Means (n = 5) for each parameter (± standard error) marked with different letters are significantly different according to Duncan’s multiple range tests (Level 0.05)

The degree of membrane damage may be a sign of how the plant is reacting to inappropriate circumstances. In this context, membrane injury was calculated and its results revealed a significant upsurge in the injury of membranes with increasing PEG concentrations from 15 at 1% PEG to 50 at 3% PEG. It is well established that water stress can compromise the stability of plant cell membranes [2, 3]. This disruption is largely attributed to the overproduction of ROS under stress conditions. At elevated levels, ROS, particularly H_2_O_2_, induce oxidative damage, primarily by breaking down the lipid components of membranes. Additionally, stress-triggered surges in superoxide anion levels have been linked to increased membrane fluidity, which indirectly reduces membrane stability [57]. Supporting this notion, Fan et al. [58] reported that PEG-induced drought stress in cucumber seedlings led to excessive accumulation of ROS, including H_2_O_2_ and superoxide radicals, along with a significant rise in MDA content. MDA, a marker of lipid peroxidation, can react with free amino groups in proteins and with membrane phospholipids, initiating ethylene production and causing alterations in membrane properties [59]. In a related study, Mickky [60] demonstrated that water stress reduced the phospholipid content in broad bean seedlings, resulting in pronounced membrane leakage, increased lipid peroxidation, and a significant decline in MSI.

Regarding the MSI values with FWE priming, its percent surged in FWE primed seedlings. Also, it is obvious that the membrane injury decreased from 15, 30 and 50 in PEG (at 1, 2 and 3%) treated seeds to 7, 18.3 and 38.4 due to FWE priming (Table 5). Fish waste contains a rich array of beneficial compounds, including amino acids, peptides, and micronutrients [12], which collectively contribute to improved plant resilience under stress conditions. Moreover, the nutrients and bioactive compounds present in fish waste promote the accumulation of osmoprotectants like proline, which help maintain cell turgor and membrane integrity during water deficit conditions [61].

### Priming with FWE attenuated PEG‑induced oxidative stress (H_2_O_2_ and MDA) in cucumber seedlings

H_2_O_2_ levels in drought-stressed cucumber seedlings rose by 2-fold relative to controls (Fig. 2), signifying oxidative stress due to impaired ROS scavenging systems. Moreover, in the current investigation, 2 and 3% PEG induced a significant accumulation of MDA in cucumber seedlings, with levels increasing by 2 and 3-fold compared to well-watered controls (Fig. 2). This surge in MDA and H_2_O_2_ is indicative of oxidative stress, as drought disrupts electron transport chains, leading to ROS overproduction and subsequent attack on polyunsaturated fatty acids in cell membranes [62, 63]. It could be because oxidative damage from water stress generated free radicals, which in turn triggered lipid peroxidation, which in turn generated MDA in the plant [64, 65].

However, seedlings primed with FWE exhibited a reduction in MDA and H_2_O_2_ content under normal and PEG induced drought compared to corresponding control plants highlighting its role in enhancing ROS detoxification and preserving membrane integrity. This reduction may stem from FWE-induced activation of antioxidant enzymes and non-enzymatic antioxidants (shown below). Additionally, FWE’s organic compounds such as chitin derivatives and amino acids (e.g., proline, glycine betaine) [12, 66] may act as osmolytes, stabilizing cellular structures and reducing ROS generation under drought.

### Priming with FWE regulated osmoprotectant accumulations (proline and protein) in PEG‑exposed cucumber seedlings

**Fig. 3:**
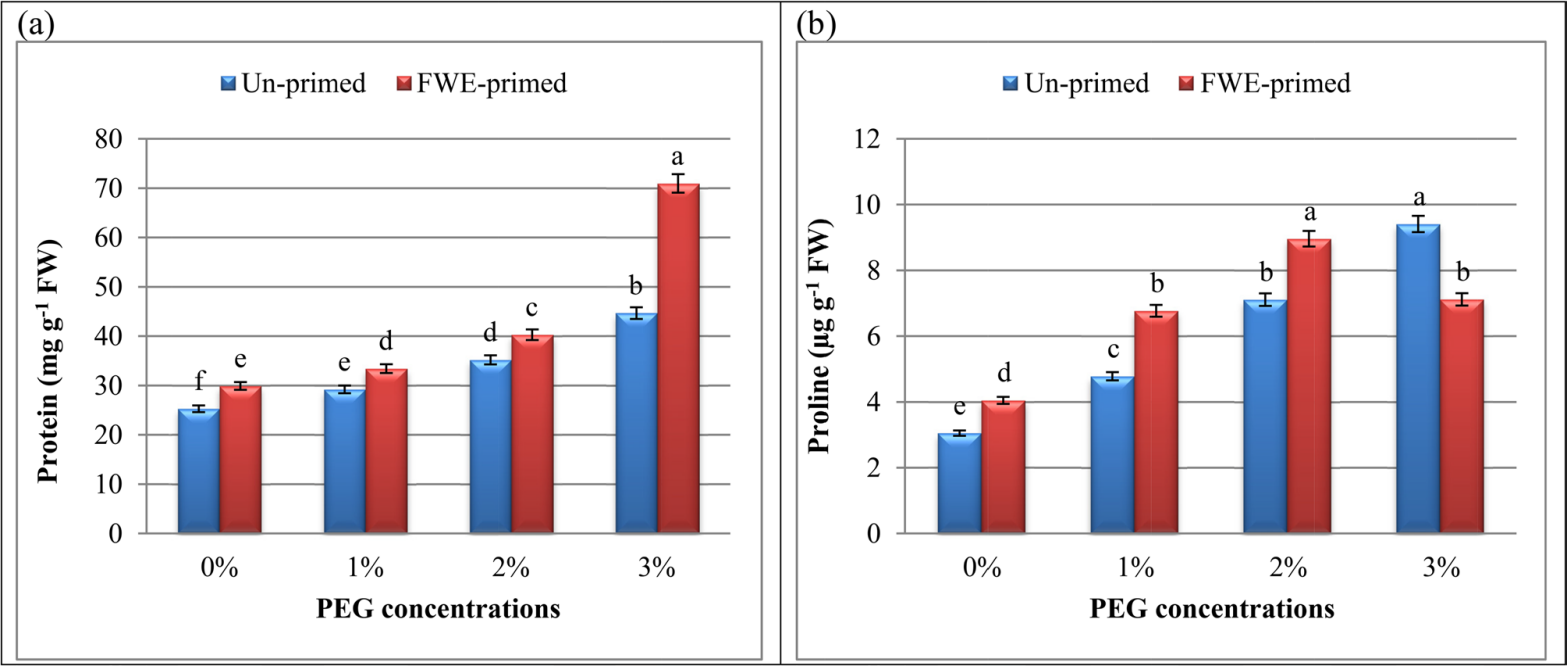
Influence of priming with fish waste extract (FWE) on protein (**a**) and proline (**b**) content of cucumber seedlings under drought stress (different polyethylene glycol: PEG). Across all treatments, data are means (*n* = 5) ± standard errors (error bars), columns followed by the same letters do not differ statistically by Duncan’s multiple range tests (Level 0.05)

Different PEG concentrations caused a noticeable rise in protein and proline (Fig. 3) contents in cucumber seedlings. Moreover, these osmoprotectants contents were greatly improved upon FWE treatment. It’s worth noting that protein and proline content tended to accumulate more in cucumber seedlings primed with FWE under different PEG conditions. The nitrogen-rich composition of FWE, containing amino acids such as glutamate and arginine, may act as substrates for protein synthesis while enhancing nitrogen assimilation pathways [67].

A recent study by Metwally et al. [12] indicated that the addition of fish waste resulted in a notable rise in the protein content of onion shoots. This could be explained by the fact that fish waste contains large quantities of muscles, which are made up of structural myofibrillar and sarcoplasmic proteins. Additionally, certain amino acids including lysine, valine, and phenylalanine are found in fish waste. These amino acids and protein, which are obtained from fish waste, are two of the main types of nitrogen that plants utilize to thrive.

Proline levels in drought-stressed seedlings increased sharply by 2-fold relative to controls (Fig. 3), a hallmark response to osmotic stress that stabilizes cellular membranes and scavenges ROS [68]. FWE priming further amplified proline accumulation under drought, surpassing levels in non-primed stressed plants at 2% PEG. Elevated proline likely contributed to improved membrane integrity and hydration, aligning with studies where organic amendments boost osmolyte synthesis to counteract water stress [2, 69].

### Priming with FWE boosted the secondary metabolites (total phenolic content; TPC and total flavonoid content; TFC) in PEG‑treated cucumber seedlings

**Fig. 4:**
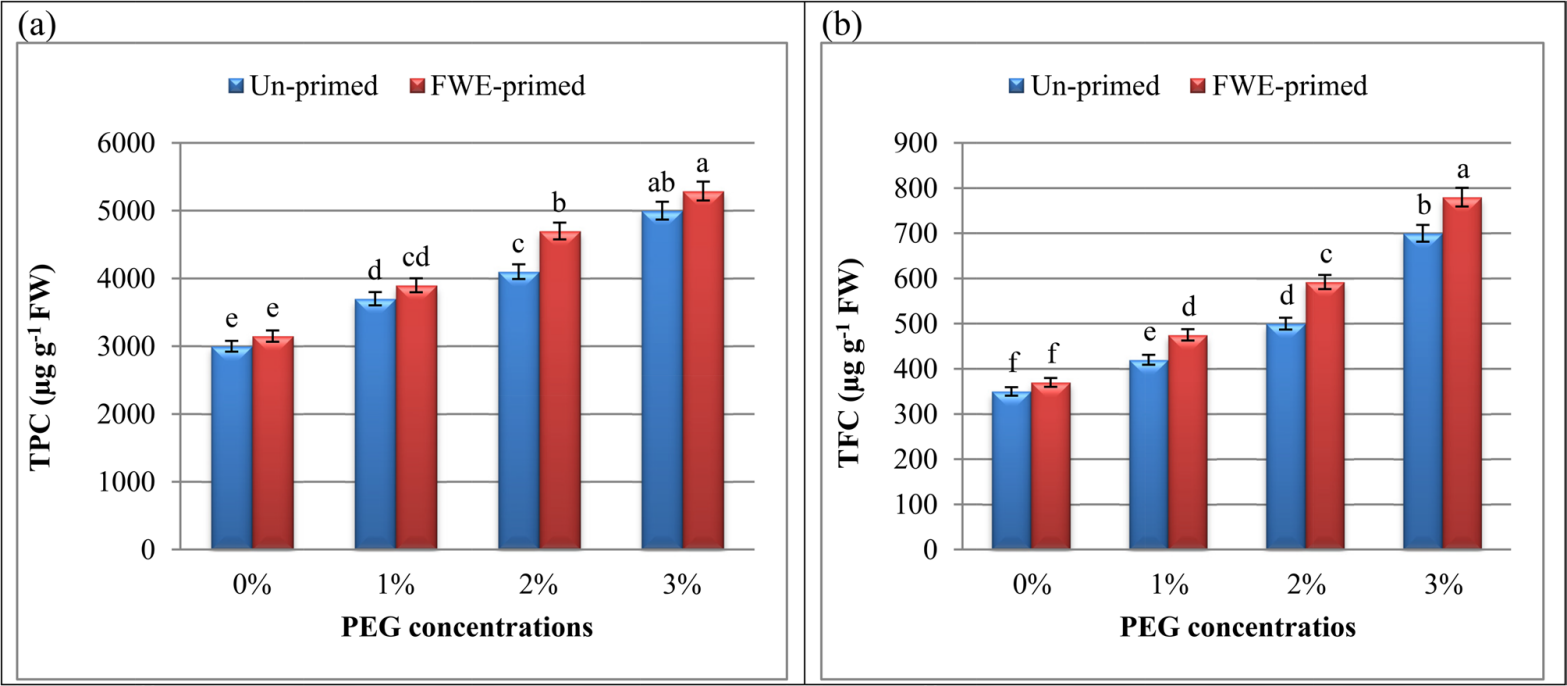
Influence of priming with fish waste extract (FWE) on (**a**) TPC: total phenolic content and (**b**) TFC: total flavonoid content of cucumber seedlings under drought stress (different polyethylene glycol: PEG). Across all treatments, data are means (n = 5) ± standard errors (error bars), columns followed by the same letters do not differ statistically by Duncan’s multiple range tests (Level 0.05)

Antioxidant metabolites are well-known to include phenolic chemicals, particularly the flavonoid subclass [70]. TPC and TFC levels were measured in the control, water-stressed and FWE-primed seedlings. TPC and TFC were increased significantly with increasing PEG concentrations (Fig. 4), but a significantly greater accumulation for both of them was detected in FWE-treated seeds relative to that of the control plants. A study by Abdelhameed et al. [2] showed an observable increase in TPC and TFC in in drought-stressed malva plants. It was well known that phenolics help plants maintain their structural integrity and protect them from stress [71], aligning with the activation of phenylpropanoid pathways under ROS stress [72].

FWE priming further elevated these metabolites surpassing drought-stressed levels. The high amino acid and other phytoconstituent (Tables 2 and 3) in FWE may act as signaling molecules, stimulating secondary metabolite biosynthesis. These compounds not only neutralize ROS but also reinforce cell walls, enhancing drought tolerance [73]. Plants use phenolic compounds as signaling molecules as part of their defense system. Increased phenolic compound production helps plants resist oxidative stress because these compounds act as water-soluble, non-enzymatic antioxidants that limit ROS and free radicals [74].

### Priming with FWE associated with antioxidant enzymes activity and total antioxidant capacity (TAC) in PEG‑stressed cucumber seedlings

**Fig. 5:**
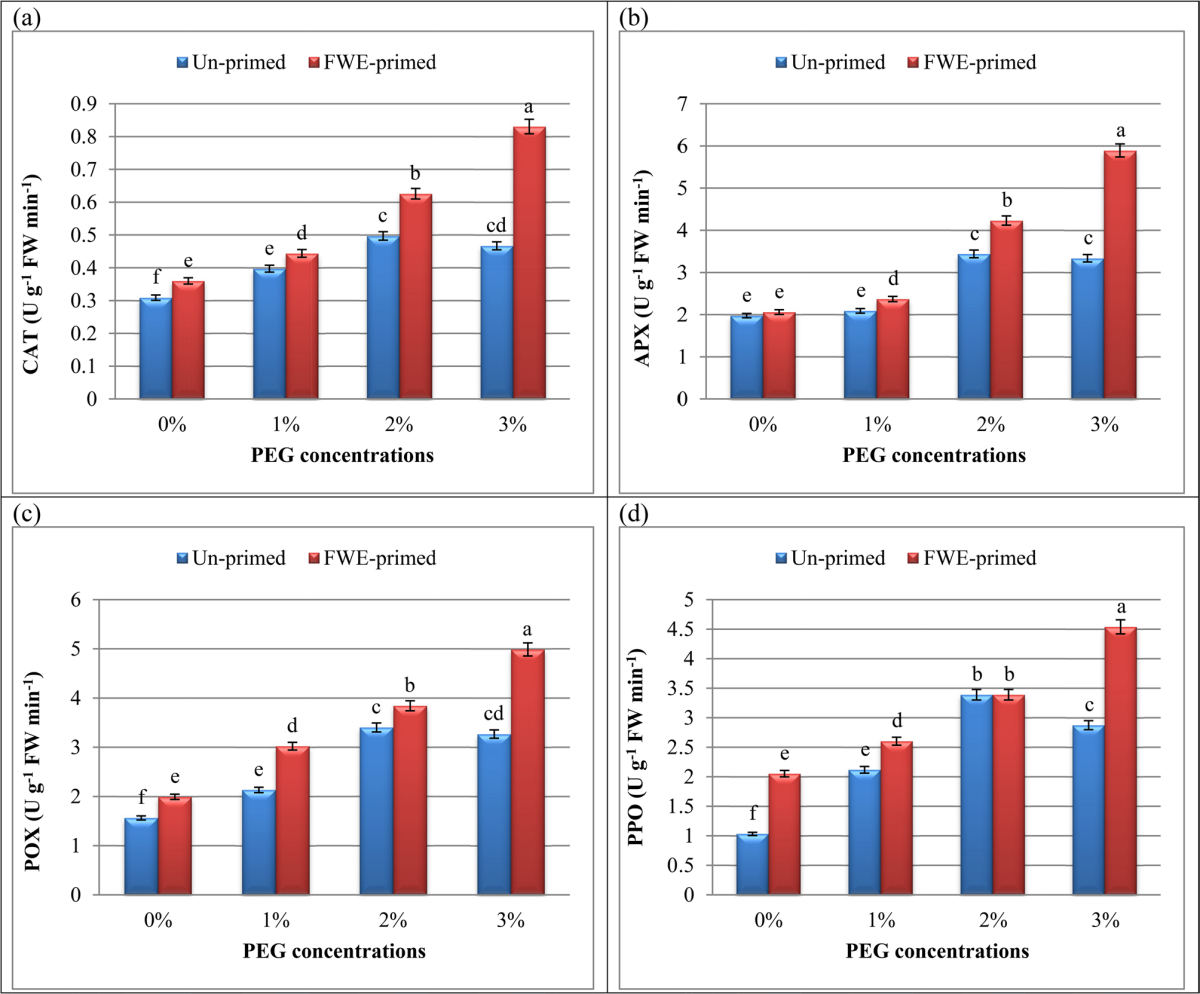
Influence of priming with fish waste extract (FWE) on the antioxidant enzymes activity [(**a**) catalase: CAT, (**b**) ascorbate peroxidase: APX, (**c**) peroxidase: POX and (**d**) polyphenol oxidase: PPO] of cucumber seedlings under drought stress (different polyethylene glycol: PEG). Across all treatments, data are means (n = 5) ± standard errors (error bars), columns followed by the same letters do not differ statistically by Duncan’s multiple range tests (Level 0.05)

Plants naturally produce antioxidants to protect themselves from oxidative stress [75, 76]; the most significant of these are glutathione reductase (GR), polyphenol oxidase (PPO), ascorbate peroxidase (APX), peroxidase (POX), catalase (CAT), and superoxide dismutase (SOD). Based on the findings displayed here **(**Fig. 5**)**, CAT, POX, APX and PPO as antioxidant enzymes were impacted by FWE and different PEG application. Drought (1 and 2% PEG) triggered a notable rise in CAT, POX, APX and PPO activities, reflecting ROS scavenging efforts. However, 3% PEG highlighted significant decrease in all measured antioxidant enzymes. In line with the present results, enhanced activity of POX, PPO and APX was reported in wheat seedlings [48, 77] and soybean plants [3] as a stress acclimation strategy. CAT is capable of breaking down H_2_O_2_ into H_2_O and O_2_, whereas POX breaks down H_2_O_2_ by oxidizing co-substrates like phenolic compounds. Alternatively, H_2_O_2_ can be scavenged by the ascorbate-glutathione cycle, which includes the reduction of glutathione by GR and the oxidation of ascorbate by APX [78].

Most interestingly, our outcomes displayed in Fig. 5 show that FWE-primed seedlings exhibited elevated antioxidant enzyme activities (approximately 3.5-fold for POX and 3-fold for CAT), consistent with a strengthened defense response. Comparable increases were also observed for APX and PPO (2.5-fold and 2.4-fold, respectively), suggesting that FWE priming is associated with enhanced enzymatic ROS-scavenging capacity. These findings align with reports from other biostimulant studies, highlighting the potential of FWE to stimulate plant antioxidant responses. This synergistic effect likely reduces oxidative damage, corroborating findings where organic priming agents bolster stress resilience. Mehla et al. [79] stated that during stress, antioxidants (APX, POX, and CAT) protect the plant somewhat and deal with scavenging ROS, H_2_O_2_, and MDA.

Regarding TAC content, PEG-induced drought revealed a significant increase in TAC (Table 5), reflecting the seedlings’ compensatory response to oxidative damage via enzymatic (CAT, POX, APX and PPO) and non-enzymatic (e.g., phenolics, flavonoids and proline) antioxidants. FWE-primed seedlings exhibited a more pronounced rise in TAC, suggesting synergistic effects between FWE-derived antioxidants and endogenous defense systems. The extract may directly contribute to ROS neutralization while stimulating phenylpropanoid pathways to enhance phenolic biosynthesis [80]. Such dual mechanisms underscore FWE’s potential to fortify antioxidant defenses, a critical trait for drought resilience.

Moreover, Table 6 showed that the two-way ANOVA interaction between FWE and PEG stress was statistically significant for some parameters (germination %, radicle length, seedling total length, VI-II, RWC, MDA, proline, protein and TAC), indicating a synergistic effect of FWE in mitigating drought-induced damage. Notably, the FWE × PEG interaction improved germination, RWC, protein, proline, enzymatic antioxidants and TAC. This suggests that FWE may play a role in enhancing cellular hydration and membrane integrity also, modulating oxidative stress responses. The enhanced performance of FWE-primed seedlings under PEG-induced drought suggests that this treatment could be harnessed as a biostimulant approach to strengthen early seedling vigor and drought adaptability in crops.

**Table 6:** Statistical evaluation of FWE priming and PEG stress interactions using two-way ANOVA

Parameters	FWE priming			PEG stress			FWE priming × PEG stress		
	F	Sig.	Partial Eta Squared (η^2^)	F	Sig.	Partial Eta Squared (η^2^)	F	Sig.	Partial Eta Squared (η^2^)
G%	210.70***	0.000	0.929	248.02***	0.000	0.979	91.10***	0.000	0.945
Shoot length	9.95**	0.006	0.384	33.30***	0.000	0.862	1.46ns	0.263	0.215
Radicle length	8.14*	0.012	0.337	34.70***	0.000	0.867	3.32*	0.047	0.383
Seedling total length	9.24**	0.008	0.366	32.92***	0.000	0.861	2.52*	0.031	0.419
Seedling FW	24.47***	0.000	0.605	26.42***	0.000	0.832	1.63ns	0.222	0.324
Seedling DW	3.40ns	0.084	0.175	16.67***	0.000	0.759	1.82ns	0.184	0.255
VI-I	239.10***	0.000	0.937	301.01***	0.000	0.983	1.12ns	0.370	0.174
VI-II	157.50***	0.000	0.908	180.90***	0.000	0.971	3.69*	0.034	0.409
RWC	7.73*	0.013	0.326	5.28*	0.010	0.498	5.42**	0.009	0.504
WSD	62.47***	0.000	0.796	170.82***	0.000	0.970	8.254**	0.002	0.607
MSI	38.01***	0.000	0.704	172.49***	0.000	0.970	0.65ns	0.596	0.108
MDA	693.80***	0.000	0.977	454.01***	0.000	0.988	19.80***	0.000	0.788
H _2_O_2_	9.52**	0.007	0.374	167.67***	0.000	0.969	0.403ns	0.753	0.070
Proline	25.48***	0.000	0.614	306.50***	0.000	0.983	62.70***	0.000	0.922
Protein	172.60***	0.000	0.915	309.30***	0.000	0.983	49.98***	0.000	0.904
TAC	111.50***	0.000	0.875	303.29***	0.000	0.983	15.07***	0.000	0.738
TPC	15.70**	0.01	0.496	162.70***	0.000	0.960	1.67ns	0.214	0.238
TFC	36.98***	0.000	0.698	257.80***	0.000	0.980	2.47ns	0.100	0.316
CAT	74.20***	0.000	0.823	307.58***	0.000	0.983	17.90***	0.000	0.770
POX	117.60***	0.000	0.880	351.90***	0.000	0.985	14.30***	0.000	0.729
APX	69.09***	0.000	0.812	474.57***	0.000	0.989	34.99***	0.000	0.868
PPO	209.70***	0.000	0.929	359.70***	0.000	0.985	9.79***	0.001	0.647

*P ≤ 0.05; **P ≤ 0.01; ***P ≤ 0.001; ns, non-significant effect

### Correlation between parameters, hierarchical clustering (HC), and principle component analysis (PCA) scatter plot

**Fig. 6:**
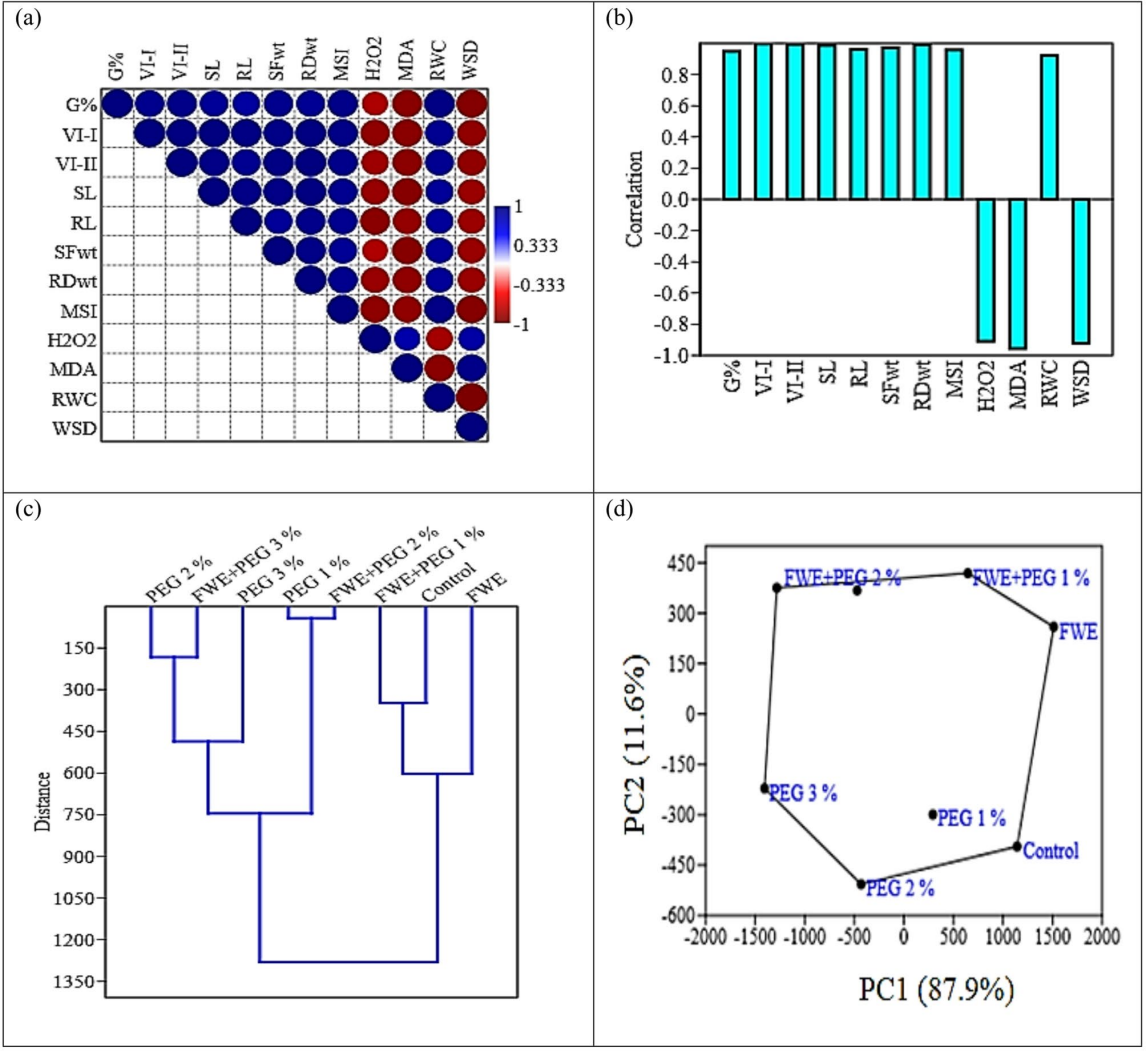
Comprehensive data analysis of the effect of FWE on PEG-induced drought stress in cucumber seedlings using correlation bivariate analysis (**a** and **b**) and multivariate analysis: clustering (**c**) and scatter plot of principal component analysis based on the variance-covariance matrix (**d**). *Note, G%: germination percentage, VI-I: vigor index-I, VI-II: vigor index-II, SL: shoot length, RL: radicle length, SFW: seedling fresh weight, SDW: seedling dry weight

To explore the interrelationships among measured parameters and used treatments, both bivariate and multivariate analyses were conducted. Bivariate correlation analysis (Fig. 6a, b) was performed to examine pairwise relationships between each of two variables. Pearson correlation analysis revealed strong positive relationships (blue color) among germination parameters (G%, VI-I, VI-II, SL, RL, SFW and SDW), RWC and MSI. These parameters were inversely correlated (red color) with stress indicators such as WSD, H_2_O_2_ and MDA.

Moreover, to evaluate the interrelationships among the used treatments, multivariate analyses, including HC and PCA, was further done. The hierarchical clustering dendrogram **(**Fig. 6c**)** grouped treatments into two different clusters. This separation highlights the ameliorative effect of FWE, which shifted the profile of PEG-stressed plants closer to that of the unstressed control. In the PCA scatter plot **(**Fig. 6d**)**, prior to PCA, all variables were standardized using z-scores. The analysis was based on the variance-covariance matrix. The first two principal components (PC1 and PC2) accounted for 87.9% and 11.6% of the total variance. This high cumulative variance indicates that most of the differences among treatments can be reliably captured in a two-dimensional plot. The PCA scatter plot clearly separated PEG-stressed cucumber seedlings from the control and FWE-treated groups. However, FWE + PEG treatments clustered closer to the control group, suggesting effective mitigation of PEG-induced disturbances. These findings support the hypothesis that FWE acts as a potent biostimulant, enhancing drought resilience in cucumber seedlings by modulating germination and oxidative stress parameters. The combined use of HC and PCA provided robust multivariate confirmation of treatment effects, highlighting the biological significance of fish waste-derived compounds in sustainable agriculture under abiotic stress.

**Fig. 7:**
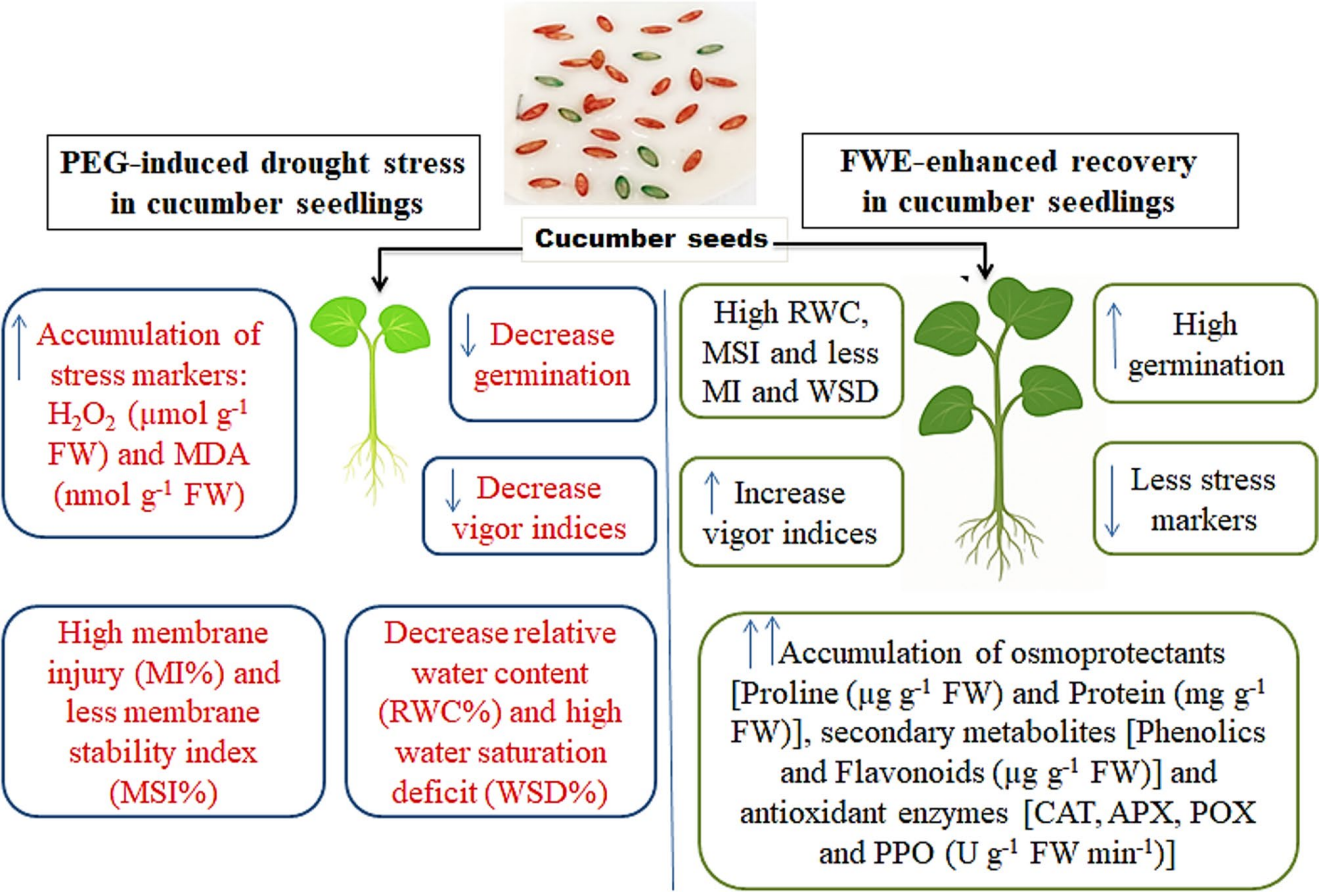
Graphical summary of the effects of PEG-induced drought and FWE-enhance recovery in cucumber seedlings

Collectively, Fig. 7 provides a comprehensive overview of how drought stress, simulated by PEG, affects cucumber seedlings, and how these effects are modulated by the application of FWE. Under drought conditions, cucumber seedlings exhibited reduced shoot and root growth, lower RWC and MSI, and elevated levels of oxidative stress markers (both MDA and H_2_O_2_). However, priming with FWE significantly alleviated these negative effects by enhancing water retention, promoting antioxidant enzyme activities (POX, PPO, CAT, APX), and restoring proline and proteins, and secondary metabolites. This illustration (Fig. 7) summarizes the dual role of drought as a stressor and FWE as a biostimulant and underscores the potential of FWE as a sustainable strategy to enhance stress resilience in cucumber plants.

## Conclusions

Fish waste priming attenuates polyethylene glycol-induced drought stress, via enhanced proline-mediated osmoprotection, and amplifies antioxidant capacity in cucumber seedlings. Fish waste extract (FWE) serves as a cost-effective, and environmentally friendly priming agent that strengthen plant tolerance against abiotic stress, offering promising implications for agricultural practices in drought-prone regions. This approach aligns with sustainable agriculture by valorizing fish waste, reducing synthetic inputs, and promoting circular economies. Future studies should explore field applications, optimal FWE concentrations, and long-term soil health impacts and scalability of this technique under varying environmental conditions. Additionally, exploring the molecular and genetic mechanisms underlying the enhanced stress response could provide deeper insights into how organic waste priming activates defense pathways.

## Data Availability

The relevant datasets supporting the results of this article are included within the article.

## Abbreviations

APX: Ascorbate peroxidase
CAT: Catalase
DW: Dry weight;
FWE: Fish waste extract
FW: Fresh weight
GR: Glutathione reductase
MDA: Malondialdehyde
MSI: Membrane stability index
POX: Peroxidase
PPO: Polyphenol oxidase
ROS: Reactive oxygen species
TAC: Total antioxidant capacity
TFC: Total flavonoid content
TPC: Total phenolic content
H_2_O_2_: Hydrogen peroxide
MI: Membrane injury
MSI: Membrane stability index
RWC: Relative water content
SOD: Superoxide dismutase
WSD: Water saturation deficit
